# What can we learn from Israel’s rapid roll out of COVID 19 vaccination?

**DOI:** 10.1186/s13584-021-00441-5

**Published:** 2021-01-26

**Authors:** Martin McKee, Selina Rajan

**Affiliations:** Department of Health Services Research and Policy, London School of Hygiene & Tropical Medicine, 15-17 Tavistock Place, London, WC1H 9SH UK

**Keywords:** COVID-19, Israel, Vaccination, Governance

## Abstract

Israel has led the world in rolling out its COVID-19 vaccination program. This experience provides lessons that others can learn from. It is, however, necessary to consider some national specificities, including the small size of the country, its young population, and the political imperative to drive this program forward. Israel also has a number of other advantages, including a strong public health infrastructure. The lessons that can be learnt include the importance of coordinating delivery mechanisms with the inevitable prioritisation of groups within the population, timely deployment of a skilled cadre of health workers, a recognition that not everyone in the population shares in the benefits of digital connectedness, the need to reach out to disadvantaged groups, based on an understanding of the barriers that they face, and the importance of placing COVID-19 vaccination within a comprehensive response to the pandemic.

## What worked in Israel and why?

As governments across the world race to roll out vaccination programmes against COVID-19, many are looking to Israel to understand how it has led the way, administering a first dose of the Pfizer-BioNtech vaccine to over 1 in 10 Israelis within 2 weeks of its approval [1]. Meanwhile, other countries, including the United States and many in Europe, are struggling to get their vaccination programs off the ground, with many accounts of doses going to waste from missed appointments or dosing challenges.

So what can we learn from the Israeli experience? In the accompanying paper, Rosen and colleagues set out a series of reasons why Israel has been so successful [2]. In this brief commentary, and drawing on the experiences of European countries in the COVID-19 response so far, we seek to draw some lessons.

As Rosen and colleagues note, Israel has some intrinsic advantages. With only 9.3 million people, it requires only a very small share of the world production of vaccine. It also has a relatively young population, making it easier to deliver a policy that prioritises older people. Also, its population density is relatively high, avoiding the need for very complex logistical arrangements to reach outlying areas. Yet, some other countries also have these advantages, to a greater or lesser degree, and they have not been so successful.

One obvious factor in Israel’s success has been the presence of a mass vaccination plan. It should be obvious that the implementation of a mass vaccination programme is a complex task, both in the normal meaning of the term “complex” and in its particular use in systems theory. While other countries focused initially on the acquisition of the stocks of vaccine, it is now apparent that many failed to understand the importance of putting in place all of the structures and processes necessary to move the vaccines from warehouses into people’s arms. As we have described elsewhere [3], this requires a series of interconnected subsystems, including those for maintaining an accurate register of the population, distributing the vaccine to facilities where it can be administered, with the added complication of maintaining a cold chain, ensuring that there are adequate numbers of trained staff to administer it, and mechanisms to identify differences in uptake within the population and responding to them. Crucially, these elements of a complex system are not self-organising. Somebody needs to be in charge with a clear vision of what they want to achieve. While this seems to be the case in Israel, it appears to have been lacking in many other countries or developed after vaccine delivery started, as was the case in the UK [4].

In reflecting why Israel seems to have got it right, it is necessary to consider some national specificities. We cannot avoid the observation by Israeli commentators that the Israeli Prime Minister Benjamin Netanyahu, facing yet another uncertain election, has his own political reasons for wanting to demonstrate success [5, 6]. While Israel seemed to do well in the early stages of the pandemic, it was unable to maintain this situation and has since experienced two large waves of infection. The ability to claim responsibility for a very successful vaccination programme has obvious attractions. In contrast, in some other countries that have fared much worse, political leaders have been disengaged, in denial, or have actively rejected scientific evidence [7].

A second consideration is the high degree of preparedness, not just for a pandemic, but for other threats to health. Like the Republic of Korea [8], Israel has considerable experience with mobilisation of the population in time of crisis. The infrastructure necessary for this to happen offers governments scope to act in ways that are denied to others that lack such organisation and capacity.

Neither of these factors are especially generalisable. So, what can others take from the Israeli experience in vaccination scale up?

## Lessons for other countries

First, given the global shortage of vaccines, which is inevitable, and given the magnitude of the task of scaling up production of a completely new product, it is necessary to prioritise groups within countries. Many countries have done so, typically focusing on older people, who are at greater risk of dying, and health or social care workers. Some, such as France, have also prioritised those in public-facing jobs, such as public transport workers and teachers. However, Israel has taken this a step further, in a way that some countries have failed to do, by linking this prioritisation to different delivery systems. Thus, different organisations are responsible for each of the four priority groups in the Israeli programme. Those over 60 or with pre-existing medical conditions were covered by the four non-profit health plans, which hold information on each individual’s medical history. Nursing home residents were covered by the national emergency services organisation Magen David Adom. Health workers were vaccinated by the organisations that employed them. Again, Israel has an advantage that is not available to some other countries, in that it has very well developed information systems [9], so that the different organisations involved in vaccination can identify those for whom they are responsible and follow them up as necessary. Notably, Israel is one of the few countries with a comprehensive population-based childhood web-based immunization registry. The platform of the national registry was rapidly adapted to the COVID-19 vaccine campaign, taking advantage of the single unique identifier for each Israeli resident that is used in all health care facilities. The registry also allows follow up and assessment of post vaccination adverse events as well as providing real-world vaccine effectiveness data.

Second, every country requires a trained workforce to deliver the vaccination programme. Again, Israel has the advantage of a cadre of well-trained community nurses with long experience in vaccination. Other countries have had to divert health professionals from acute care, a highly undesirable necessity at a time when many health systems are being overwhelmed with patients suffering from the acute effects of Covid 19. Both of these considerations emphasise the importance of investing in health infrastructure, whether in the form of trained staff, facilities, or information technology in normal times, so that there is adequate capacity when a crisis strikes [10].

A third observation in the Israeli experience was the problems that arose in booking appointments in the initial days of the campaign. Like many other countries, they relied, to a considerable extent, on the use of online scheduling and call centres. However, while these approaches have proven very successful in other aspects of modern life, such as online shopping or travel reservations, it is easy to forget how those who take advantage of these opportunities are not representative of the population as a whole, or of those in the priority groups. The COVID-19 pandemic has highlighted the existence, in many wealthy countries, of large groups of people who are in effect excluded from the digital world [11], for example due to lack of internet connectivity or language problems in communicating with health providers by phone [12]. Until this is addressed at some time in the future, it will still be necessary to make use of the traditional forms of interaction between the individual and the state.

Fourth, as Rosen and colleagues note, there have been particular challenges in reaching some groups. While some earlier challenges in achieving vaccine uptake in the ultra-orthodox Jewish community have been addressed, uptake was also initially low in Israeli Arab areas and it is taking longer to address that challenge [13]. Disadvantaged communities in all countries have borne the brunt of the pandemic, with higher rates of infection [14] and worse outcomes [15]. They have also suffered most from the economic and social consequences of responses. In essence, COVID-19 has shone  a light on existing fractures in societies. Consequently, any failure to reach these groups will exacerbate existing inequalities. Consequently, a comprehensive vaccination strategy must include a system to monitor uptake in different groups, something that is impossible in the many countries that fail to collect data on ethnicity and occupation, and to understand why some are excluded. This will require mixed methods research coupled with co-creation of contextually appropriate feasible solutions [16].

Finally, while this commentary and the accompanying paper focus on vaccination, this is only one element of a comprehensive COVID-19 strategy. It must be accompanied by measures to drive down levels of circulating infection and keep them low with well functioning find, test, trace, isolate and support systems [17]. Crucially, while the existing vaccines have been shown to reduce the risk of people becoming ill, they may be less successful in preventing infection and onward transmission. There is a danger that politicians will see vaccination as a magic bullet that will, somehow, make the threat from COVID-19 go away. It will not, and we must not ignore the risk of potential vaccine escape mutations emerging, something that is more likely if high levels of virus continue to circulate.

It is also critical that any vaccine rollout is accompanied by appropriate post-manufacturing surveillance to document the real world efficacy of the different vaccines in different patient groups [18]. This is especially important in Israel where any issues will become apparent sooner than elsewhere and where the information system, mentioned earlier, facilitates this. Thus, as expected based on the Phase 3 trial [19], a single dose of the vaccine offers only partial protection, and only after about 12 days. Those who have received the first dose should not count on protection, and indeed, in Israel 17% of seriously ill persons as of mid-January 2021 had received a single dose of vaccine [20]. Even after a second dose, individuals should maintain precautions against exposure until the level of circulating virus in the community falls to very low levels.

## Conclusion

There is much to be learned from the experiences of those in the forefront of rolling out vaccination programmes, such as Israel, but this should include a whole system perspective that takes account of differences in context.

## Data Availability

Not applicable.
